# Novel Ensemble Approach with Incremental Information Level and Improved Evidence Theory for Attribute Reduction

**DOI:** 10.3390/e27010094

**Published:** 2025-01-20

**Authors:** Peng Yu, Yifeng Zheng, Ziwen Liu, Baoya Wei, Wenjie Zhang, Ziqiong Lin, Zhehan Li

**Affiliations:** 1 School of Computer Science, Minnan Normal University, Zhangzhou 363000, China; g2022062008@stu.mnnu.edu.cn (P.Y.); zyf@mnnu.edu.cn (Y.Z.); g2023062007@stu.mnnu.edu.cn (Z.L.); zhan0300@ntu.edu.sg (W.Z.); lzq1142@mnnu.edu.cn (Z.L.); lzh_mnnu@163.com (Z.L.); 2 Key Laboratory of Data Science and Intelligence Application, Fujian Province University, Zhangzhou 363000, China

**Keywords:** machine learning, attribute reduction, information theory, evidence theory

## Abstract

With the development of intelligent technology, data in practical applications show exponential growth in quantity and scale. Extracting the most distinguished attributes from complex datasets becomes a crucial problem. The existing attribute reduction approaches focus on the correlation between attributes and labels without considering the redundancy. To address the above problem, we propose an ensemble approach based on an incremental information level and improved evidence theory for attribute reduction (IILE). Firstly, the incremental information level reduction measure comprehensively assesses attributes based on reduction capability and redundancy level. Then, an improved evidence theory and approximate reduction methods are employed to fuse multiple reduction results, thereby obtaining an approximately globally optimal and a most representative subset of attributes. Eventually, using different metrics, experimental comparisons are performed on eight datasets to confirm that our proposal achieved better than other methods. The results show that our proposal can obtain more relevant attribute sets by using the incremental information level and improved evidence theory.

## 1. Introduction

With the development of intelligent technology, the scale of data in practical applications is increasing daily. However, it is hard to effectively extract essential information from complex internal relationships in data for decision-making [1]. Therefore, the attribute reduction method is proposed to solve the above problem [2,3].

The core idea of attribute reduction is to select a subset of attributes with the most significant classification ability. Rough set theory has been widely used in attribute reduction, as proposed by Pawlak [4]. It constructs the upper- and lower-approximations, which are based on the equivalence relationship, to describe the relationships in data. However, the division of the equivalence relations is too strict, so the obtained attribute subset overly relies on the given data, which makes the classifier prone to over-fitting. On this basis, researchers proposed the probabilistic rough set [5], neighborhood rough set [6], and fuzzy rough set [7].

According to the adopted strategies, attribute reduction can be divided into embedded-based, wrapper-based, and filter-based approaches [8]. The embedded-based methods integrate the reduction process into the model training process. For the wrapper-based method, the classifier’s performance is utilized as the criterion to judge the quality of the attribute subset. However, the above two methods rely too much on the classifier, resulting in a long reduction time and reduced generalization ability. The filter-based method can be viewed as data pre-processing independent of the classification model. It analyzes the intrinsic correlation between data to obtain attribute subsets, which can effectively reduce the time complexity of the model.

However, attribute reduction usually employs a single metric criterion to evaluate attribute effectiveness. That is, it focuses on the classification ability of attributes without considering the redundancy between attributes and existing attribute subsets [9]. The intuitive manifestation of the above problem is that the reduction rate is low, which results in the reduction set being entirely consistent with the original attribute set for some datasets. Therefore, reducing the internal redundancy of the reduction attribute set can effectively improve the reduction rate.

It should be noted that the dataset is usually divided into disjoint training sets and test sets simultaneously in model construction [10]. Therefore, the obtained data subset is localized and cannot represent the characterization of the original data space. It makes the model’s performance closely related to a single test set, and the classification ability is significantly different and not representative. Evidence fusion is one of the effective methods for addressing the above problem [11,12]. In recent years, various improvements based on evidence fusion theory have been proposed, including time series complexity measurement-based methods [13], fractal confidence-based methods [14,15], and multi-source data fusion-based methods [16]. However, the above methods ignore the correlation between attributes in the evidence fusion process. Some researchers have made improvements in the aspect of redundancy only by using independent redundancy constraints [17,18].

Therefore, to solve the above problems, we propose in this paper an ensemble attribute reduction method (IILE) based on incremental information level and evidence theory. Firstly, the incremental information level is defined as a metric that considers the ability to judge classification and the degree of redundancy for obtaining attributes more efficiently. Secondly, multiple data subspaces are constructed through random sampling for searching multiple attribute subsets. Then, we adopt the improved evidence theory as a group fusion strategy for fusing the obtained multiple subsets in order to obtain an approximate global optimization attribute reduction set. Eventually, the approximate reduction strategy is utilized to reduce the influence of noise data on the original dataset.

The main contributions of this paper are as follows:

(1) *Correlation-redundancy measure*: The incremental level measure comprehensively evaluates the correlation between attributes, labels, and the redundancy of attribute subsets in order to obtain the most discriminative attribute reduction subset.
(2) *Ensemble learning*: The improved evidence fusion theory strategy fuses the different attribute reduction subsets obtained by multiple sample subspaces in order to obtain a global attribute reduction set and characterize the global properties of the original data space.
(3) *Approximate reduction method*: Using approximate reduction methods to reduce the impact of noisy samples in the dataset, making the results more representative.

The remaining sections are as follows: Section 2 mainly introduces the related work on attribute reduction. Section 3 illustrates the related definition of a rough set. The proposed IILE is carefully described in Section 4. Section 5 provides a detailed analysis of comparative experiments. Section 6 summarizes our proposal and future research work.

## 2. Related Work

Attribute reduction is one of the effective methods for extracting critical information from data. Pawlak proposed an attribute reduction method according to the positive region [19], which adopts the rough set to divide the data into indistinguishable relationships to select attributes. The drawback is that it is easy to cause over-fitting problems. To solve the above problem, Li et al. [20] introduced the weighting method to optimize the decision table to improve the algorithm’s performance. Zhang et al. [21] improved the traditional subgroup discovery algorithm and adopted the ensemble method to maximize the algorithm’s performance. In addition, to solve the problem of over-fitting and the high time complexity of reduction, Fang et al. [22] proposed a hyperspherical neighborhood rough set to improve the classification accuracy, which causes the size of the neighborhood radius to be adjusted adaptively according to the positive domain. Li et al. [23] utilized inter-cluster divergence to optimize the conventional rough set to address continuous data. Dai et al. [24] proposed fuzzy joint entropy based on the fuzzy rough set and information entropy to achieve attribute reduction. Kang et al. [25] observed that the conventional reduction method would cause the loss of the original attribute information. Therefore, they adopted the population algorithm to obtain the feature subset to improve classification accuracy. In addition, to solve the influence of class imbalance on reduction, Sun et al. [26] combined a population algorithm based on neighborhood tolerance mutual information to reduce the influence of class imbalance. Xia et al. [27] proposed the particle sphere neighborhood model, which has better flexibility and more comprehensive versatility for different datasets. Given the negative impacts of different labels on each other in the data, Guo et al. [28] introduced spatial similarity in the reduction process to obtain attribute reduction subsets in the same space to improve the classification performance. However, the above approaches cannot consider the redundancy between attributes, resulting in many unnecessary attributes in the final reduction results.

Nowadays, information theory can be employed to measure the internal relationship between variables. It can effectively calculate the degree of correlation and the redundancy among variables. Xu et al. [29] combined the information gain ratio with the improved genetic algorithm to significantly enhance the decision-making level. Mou et al. [30] proposed neighborhood conditional entropy for a specific class to improve the classification performance. Gao et al. [31] transformed multi-value classification into a binary classification problem and introduced weights to construct parameterized maximum entropy to improve the model’s performance. Qiu et al. [32] proposed the total correlation information coefficient to avoid the influence of hyperparameters on time complexity and shorten the overhead of attribute reduction on high-dimensional datasets. Wang et al. [33] presented the neighborhood self-information measure to enhance the attribute subset’s training effect further. Qu et al. [34] observed that samples with different labels adversely affect each other. They utilized rough mutual information to expand the intrinsic correlation of attribute subsets to reduce redundancy, thereby improving the reduction quality. However, the above approaches are performed in the sampling space, and the obtained results are locally optimal and cannot represent the characteristics of the original data space.

Therefore, we considered constructing multiple sample subspaces to obtain the approximate global optimization attribute subset. Firstly, we adopt the two quantitative values of the classification ability and redundancy degree to evaluate the correlation between attributes. In addition, we utilize improved evidence theory combined with approximate reduction as an ensemble learning strategy to measure the adverse effects of noise and obtain the approximate global optimal attribute subset. The difference between our proposal and others is shown in Table 1.

## 3. Related Definitions

This section provides a brief introduction to the relevant definitions of rough sets and information systems.

### 3.1. Rough Set

A rough set can be utilized for the attribute reduction of an information system [4]. The information system can be expressed as ℜ=(U,A,ν), where $U=\{u_{1},u_{2},\ldots ,u_{m}\}$ is a non-empty finite set $\left|U\right|=m$. $A=\{a_{1},a_{1},\ldots ,a_{n}\}$ is a non-empty finite attribute set $\left|A\right|=n$. ν can be expressed as a mapping function that maps $u_{i}$ to the value domain with respect to attribute $a_{j}$, i.e., $\nu_{a_{j}}\left(u_{i}\right)=k$.

Given ℜ=(U,A,ν), let *C* denote the conditional attribute set and *D* denote the decision attribute set. If A=C∪D and C∩D=∅, then an information system ℜ=(U,A,ν) is called a decision table, denoted as ${ℜ}_{DS}=\left(U,C,D,\nu \right)$.

### 3.2. Information Theory

Information theory can be utilized to measure the uncertainty and the correlation between multiple variables.

**Definition** **1** (**Information entropy** [35])**.** *Given ${ℜ}_{DS}=\left(U,C,D,\nu \right)$, for ∀B⊆C, the division of U with respect to B can be denoted as $U/B=\{X_{1},X_{2},\ldots ,X_{\left|U/B\right|}\}$, the information entropy of B on U is defined as follows:*

(1) $${ℑ}_{E}\left(B\right)=-\sum_{i=1}^{\left|U/B\right|}p(X_{i})\log p\left(X_{i}\right)$$

*where $p\left(X_{i}\right)=|X_{i}\left|/|U\right|$ denotes the probability that $X_{i}$ is selected.*

**Definition** **2** (**Conditional entropy** [35])**.** *Given ${ℜ}_{DS}=\left(U,C,D,\nu \right)$, the division of U with respect to condition attribute set C can be denoted as $U/C=\{X_{1},X_{2},\ldots ,X_{\left|U/C\right|}\}$, the division of U with respect to decision attribute D can be denoted as $U/D=\{Y_{1},Y_{2},\ldots ,Y_{\left|U/D\right|}\}$, and conditional entropy is defined as follows:*

(2) $${ℑ}_{CE}\left(D|C\right)=-\sum_{i=1}^{\left|U/C\right|}p(X_{i})\sum_{j=1}^{\left|U/D\right|}p\left(Y_{j}|X_{i}\right)\log p(Y_{j}\left|X_{i}\right)$$

*where $p\left(Y_{j}|X_{i}\right)=\;|X_{j}\cap Y_{j}\left|/\right|X_{i}|$ denotes the probability of $X_{i}$ given $Y_{j}$.*

**Definition** **3** (**Mutual information** [35])**.** *Given ${ℜ}_{DS}=\left(U,C,D,\nu \right)$, the division of U with respect to the condition attribute set C can be denoted as $U/C=\{X_{1},X_{2},\ldots ,X_{\left|U/C\right|}\}$, the division of U with respect to decision attribute D can be denoted as $U/D=\{Y_{1},Y_{2},\ldots ,Y_{\left|U/D\right|}\}$, and mutual information is defined as follows:*

(3) $${ℑ}_{MI}\left(C,D\right)=-\sum_{i=1}^{\left|U/C\right|}\sum_{j=1}^{\left|U/D\right|}p\left(X_{i},Y_{j}\right)\log\frac{p(X_{i},Y_{j})}{p\left(X_{i}\right)p\left(Y_{j}\right)}$$

## 4. IILE Algorithm

With increased data dimensions, the number of attribute subset combinations increases exponentially. In practical applications, mutual information can be used to measure the information contained between attributes. Notably, the mutual information between attributes possessing strong classification capability and labels tends to be significantly higher compared to attributes with weaker classification ability. Therefore, researchers consider utilizing mutual information for attribute reduction to reduce the data dimension.

**Figure 1:**
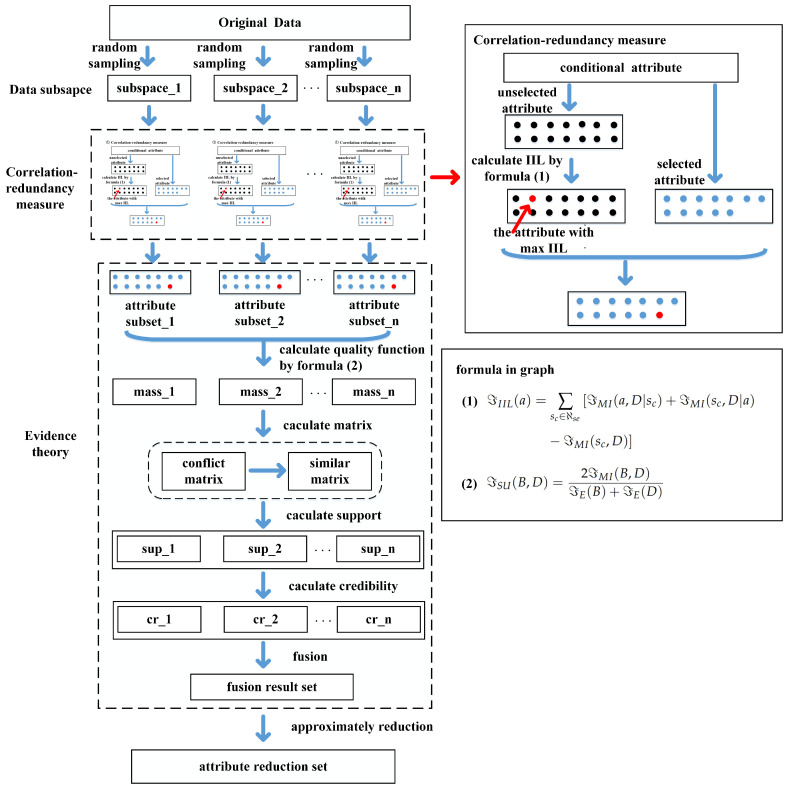
Flow chart of IILE.

In this section, we first propose the incremental information level to measure attributes. Secondly, evidence fusion theory and approximate reduction are combined to obtain the approximate global optimal attribute reduction. The model framework is shown in Figure 1.

### 4.1. Incremental Level of Information

In ${ℜ}_{DS}=\left(U,C,D,\nu \right)$, mutual information can be employed to obtain attributes with high correlation of label quickly. However, the attribute subset obtained using the above method usually has high redundancy. The degree of redundancy also increases with increased data dimensions, which decreases the reduction efficiency and the model’s performance.

The redundancy between attributes can viewed as the correlation between attributes. For any two attributes, the more similar they are, the higher the correlation between them. That is, if one has been selected, the redundancy brought about by the other will be higher than its classification ability. To this end, we propose an incremental information level to consider the redundancy measurement while measuring the correlation between attributes and labels. The specific definition is as follows:

**Definition** **4.** ***Incremental Information Level**: Given ${ℜ}_{DS}=\left(U,C,D,\nu \right)$, let ${ℵ}_{se}\subseteq C$ represent the set of selected attributes and ${ℵ}_{us}=C-{ℵ}_{se}$ represent the complementary set of ${ℵ}_{se}$. For $\forall a\in {ℵ}_{us}$, the incremental information level is denoted as follows:*

(4) $${ℑ}_{IIL}\left(a\right)=\sum_{s_{c}\in {ℵ}_{se}}\left[{ℑ}_{MI}\left(a,D|s_{c}\right)+{ℑ}_{MI}\left(s_{c},D|a\right)-{ℑ}_{MI}\left(s_{c},D\right)\right]$$

*where $s_{c}$ represents one of attributes in ${ℵ}_{se}$ and ${ℵ}_{se}$ represents the obtained attribute subset during the process of attribute reduction.*

It can be seen from Equation (4) that the incremental information level of attributes includes two parts. The former ${ℑ}_{MI}\left(a,D|s_{c}\right)$ quantifies the classification level of attribute *a* under given the condition of $s_{c}$. That is the mutual information between unselected attribute *a* and label *D*. The latter ${ℑ}_{MI}\left(s_{c},D|a\right)-{ℑ}_{MI}\left(s_{c},D\right)$ measures the additional classification ability of attribute *a* given $s_{c}$. The larger the value, the more significant the increased classification ability, and vice versa. Therefore, the incremental information level tends to select features that strongly correlate with the label and consider the influence of the selected feature set simultaneously. To provide a more intuitive demonstration of the usage of Formula (4), an example is presented for clarification.

**Example** **1.** *Given ${ℜ}_{DS}=\left(U,C,D,\nu \right)$, $U=\{u_{1},u_{2},\ldots ,u_{11}\}$, $C=\{c_{1},c_{2},\ldots ,c_{4}\}$, the final obtained reduction is denoted as P. The specific data are shown in Table 2.*

In each round, select the attribute with the maximum Incremental Information Level (IIL) and add it to the attribute reduction set *P*. In the first round, ${ℵ}_{se}=⌀$, then ${ℑ}_{IIL}\left(c_{1}\right)=0.1269$, ${ℑ}_{IIL}\left(c_{2}\right)=0.2879$, ${ℑ}_{IIL}\left(c_{3}\right)=0.1064$, ${ℑ}_{IIL}\left(c_{4}\right)=0.1310$. Therefore, in the first round, select the attribute $c_{2}$ and add it to *P*, which is, at this time, ${ℵ}_{se}=\left\{c_{2}\right\}$. In the second round, ${ℑ}_{IIL}\left(a_{1}\right)=0.3257$, ${ℑ}_{IIL}\left(a_{3}\right)=0.5122$, and ${ℑ}_{IIL}\left(a_{4}\right)=0.1310$. At this point, select $c_{3}$ to join *P*, at this time, ${ℵ}_{se}=\left\{a_{2},a_{3}\right\}$. In the third round, ${ℑ}_{IIL}\left(a_{1}\right)=2.7314$, ${ℑ}_{IIL}\left(a_{4}\right)=1.9570$, select $c_{1}$ to join *P*, then ${ℵ}_{se}=\left\{a_{2},a_{3},a_{1}\right\}$. In the fourth round, ${ℑ}_{IIL}\left(a_{4}\right)=2.5721$, choose $c_{4}$ join *P*. Finally, ${ℵ}_{se}=\left\{a_{2},a_{3},a_{1},a_{4}\right\}$, and $P=\{a_{2},a_{3},a_{1},a_{4}\}$.

For Formula (4), it has the following proposition.

**Proposition** **1.** *Given ${ℜ}_{DS}=\left(U,C,D,\nu \right)$, for ∀a∈C, ${ℑ}_{IIL}\left(a\right)\geq 0$.*

**Proof.**  $${ℑ}_{IIL}\left(a\right)=\sum_{s_{c}\in {ℵ}_{se}}\left[{ℑ}_{MI}\left(a,D|s_{c}\right)+{ℑ}_{MI}\left(s_{c},D|a\right)-{ℑ}_{MI}\left(s_{c},D\right)\right]$$

$$\sum_{s_{c}\in {ℵ}_{se}}\left[{ℑ}_{MI}\left(a,D|s_{c}\right)+{ℑ}_{MI}\left(s_{c},D|a\right)-{ℑ}_{MI}\left(s_{c},D|a\right)\right]\leq {ℑ}_{IIL}\left(a\right)$$

$$\sum_{s_{c}\in {ℵ}_{se}}{ℑ}_{MI}\left(a,D|s_{c}\right)\leq \sum_{s_{c}\in {ℵ}_{se}}\left[{ℑ}_{MI}\left(a,D|s_{c}\right)+{ℑ}_{MI}\left(s_{c},D|a\right)-{ℑ}_{MI}\left(s_{c},D\right)\right]$$

Since the value of mutual information is greater than zero, we have

$${ℑ}_{MI}\left(a,D|s_{c}\right)\geq 0$$

According to the transitivity of inequalities, we have

$$0\leq \sum_{s_{c}\in {ℵ}_{se}}{ℑ}_{MI}\left(a,D|s_{c}\right)\leq \sum_{s_{c}\in {ℵ}_{se}}\left[{ℑ}_{MI}\left(a,D|s_{c}\right)+{ℑ}_{MI}\left(s_{c},D|a\right)-{ℑ}_{MI}\left(s_{c},D\right)\right]$$

Therefore, we have

$${ℑ}_{IIL}\left(a\right)=\sum_{s_{c}\in {ℵ}_{se}}\left[{ℑ}_{MI}\left(a,D|s_{c}\right)+{ℑ}_{MI}\left(s_{c},D|a\right)-{ℑ}_{MI}\left(s_{c},D\right)\right]\geq 0$$

   □

In this paper, we propose a new attribute reduction algorithm based on the incremental information level. The specific process is shown in Algorithm 1.
**Algorithm 1** Based on incremental information level approximation (IIL).**Input**: ${ℜ}_{DS}=\left(U,C,D,\nu \right)$. **Output**: reduction set red.   1: red←∅   2: calculate the incremental information level of each unselected attribute   3: select the attribute with the maximum incremental information   4: $a_{0}\leftarrow \arg\max{ℑ}_{IIL}\left(a\right)$   5: $red\leftarrow red\cup \left\{a_{0}\right\}$   6: if ${ℑ}_{MI}\left(C,D\right)>{ℑ}_{MI}\left(red,D\right)$, go to line 7; otherwise, go to line 2   7: return to the reduction red.

For Algorithm 1, it is assumed that the number of samples is $\left|U\right|$, the number of attributes is $\left|C\right|$, the time complexity of calculating the incremental information level is $O\left(1\right)$, and the time complexity of the process of selecting attributes is $O\left(C\right)$. In the above process, the $\left|C\right|$ round needs to be repeated; the final time complexity is $O(|C{|}^{2})$.

During the practical reduction process, a sampling technique is always utilized to construct a data subspace, leading to the obtained attribute subset being locally optimal. Therefore, in this paper, we take a global perspective and adopt evidence theory to obtain an approximate globally optimal attribute subset.

The core concept of evidence fusion theory is to synthesize various pieces of evidence (attribute subsets in this paper) to obtain a comprehensive result. In the fusion process, the Minkowski distance can be used to assess the level of conflict between different items of evidence.

**Definition** **5.** ***Minkowski Distance**: Given ${ℜ}_{DS}=\left(U,C,D,\nu \right)$, for ∀a∈C, $u_{e},u_{f}\in U$, the Minkowski distance between $u_{1}$ and $u_{2}$ with respect to a is defined as follows:*

(5) $$Dis_{MD}\left(u_{e},u_{f}\right)={\left(\sum_{u_{e},u_{f},\forall a\in C}{|\nu_{a}\left(u_{e}\right)-v_{a}\left(u_{f}\right)|}^{\omega }\right)}^{\frac{1}{\omega }}$$

*where ω is any nonzero real number.*

Equation (5) is the standard formula for Minkowski distance. When ω=1, it becomes the Manhattan distance. When ω=2, it is the Euclidean distance. When ω→∞ it becomes the Chebyshev distance.

The magnitude of Minkowski’s distance indicates the conflict between two pieces of evidence. That is, the higher the value, the greater the conflict is, and vice versa. Therefore, we consider adopting Minkowski’s distance to measure the conflict distance between different attribute subsets.

**Definition** **6.** ***Evidence Conflict Distance**: Given ${ℜ}_{DS}=\left(U,C,D,\nu \right)$, ${ℵ}_{n}$.${ℵ}_{m}$ denotes two different pieces of evidence, which can be expressed as ${ℵ}_{i}=\left(a_{1},a_{2},\ldots ,a_{\left|C\right|}\right)$. The evidence conflict distance based on the Minkowski distance is denoted as follows:*

(6) $$\chi_{mn}={\left(\sum_{j=1}^{\left|C\right|}|{ℵ}_{n}\left(j\right)-{ℵ}_{m}\left(j\right){|}^{\omega }\right)}^{\frac{1}{\omega }}$$

*where ℵ(j) represents the j-th element of the evidence.*

Equation (6) is a distance formula, which satisfies non-negativity, symmetry, and the triangle inequality.

**Non-negativity**: ∀a,b, $\chi_{ab}\geq 0$
**Symmetry**: ∀a,b, $\chi_{ab}=\chi_{ba}$
**Triangle inequality**: ∀a,b,c, $\chi_{ac}\leq \chi_{ab}+\chi_{bc}$.

**Proof.**  Obviously, the non-negativity and symmetry are satisfied. Therefore, the main task is to prove the triangle inequality. For any three pieces of evidence ${ℵ}_{a},{ℵ}_{b},{ℵ}_{c}$, the evidence conflict distance between each pair of them can be expressed as follows:

$$\chi_{ac}={\left(\sum_{j=1}^{j=|C|}|{ℵ}_{a}\left(j\right)-{ℵ}_{c}\left(j\right){|}^{\omega }\right)}^{\frac{1}{\omega }}$$

$$\chi_{ab}={\left(\sum_{j=1}^{j=|C|}|{ℵ}_{a}\left(j\right)-{ℵ}_{b}\left(j\right){|}^{\omega }\right)}^{\frac{1}{\omega }}$$

$$\chi_{bc}={\left(\sum_{j=1}^{j=|C|}|{ℵ}_{a}\left(j\right)-{ℵ}_{c}\left(j\right){|}^{\omega }\right)}^{\frac{1}{\omega }}$$

According to the triangle inequality of absolute value, we have $|{ℵ}_{a}\left(j\right)-{ℵ}_{c}\left(j\right)|\leq \;|{ℵ}_{a}\left(j\right)-{ℵ}_{b}\left(j\right)|\;+\;|{ℵ}_{b}\left(j\right)-{ℵ}_{c}\left(j\right)|$. Therefore, the following equation holds.

$${\left(\sum_{j=1}^{j=|C|}|{ℵ}_{a}\left(j\right)-{ℵ}_{c}\left(j\right){|}^{\omega }\right)}^{\frac{1}{\omega }}\leq {\left[\sum_{j=1}^{j=|C|}\left(|{ℵ}_{a}\left(j\right)-{ℵ}_{b}\left(j\right)\right|+|{ℵ}_{b}\left(j\right)-{ℵ}_{c}\left(j\right)|\right)}^{w}{]}^{\frac{1}{\omega }}$$

According to the Minkowski inequality, we have

$${\left[\sum_{j=1}^{j=|C|}\left(|{ℵ}_{a}\left(j\right)-{ℵ}_{b}\left(j\right)\right|+|{ℵ}_{b}\left(j\right)-{ℵ}_{c}\left(j\right)|\right)}^{\omega }{]}^{\frac{1}{\omega }}$$

$$\leq {\left[\sum_{j=1}^{j=|C|}{\left(|{ℵ}_{a}\left(j\right)-{ℵ}_{b}\left(j\right)|\right)}^{\omega }\right]}^{\frac{1}{\omega }}+{\left[\sum_{j=1}^{j=|C|}{\left(|{ℵ}_{b}\left(j\right)-{ℵ}_{c}\left(j\right)|\right)}^{\omega }\right]}^{\frac{1}{\omega }}$$

So, we have

$$\forall a,b,c,\chi_{ac}\leq \chi_{ab}+\chi_{bc}$$

The triangle inequality holds.    □

Based on Equation (6), the normalized conflict matrix can be denoted as follows:

(7) $$MC_{mn}=\left[\begin{array}{cccc} 0 & \chi_{12} & \cdots & \chi_{1n}\\ \chi_{21} & 0 & \cdots & \chi_{2n}\\ \vdots & \vdots & \ddots & \vdots \\ \chi_{m1} & \chi_{m2} & \ldots & 0 \end{array}\right]$$

Contrary to the level of conflict, evidence support refers to the support degree of evidence supported by others. The higher the similarity with other evidence, the higher the support degree, and vice versa. That is, the more support the evidence receives, the more global the evidence is. According to Equation (6), The similarity between ℵ(j) and ℵ(i) is defined as follows:

(8) $$\kappa_{ij}=1-\chi_{ij},i,j=1,2,\ldots ,n$$

Similar to Equation (7), the normalized similarity matrix can be expressed as follows:

(9) $$MS_{mn}=\left[\begin{array}{cccc} 0 & \kappa_{12} & \cdots & \kappa_{1n}\\ \kappa_{21} & 0 & \cdots & \kappa_{2n}\\ \vdots & \vdots & \ddots & \vdots \\ \kappa_{m1} & \kappa_{m2} & \ldots & 0 \end{array}\right]$$

For $\forall {ℵ}_{i}$ its evidence support degree is defined as follows:

(10) $$\psi_{sup}\left({ℵ}_{i}\right)=\sum_{j=1,j\neq i}^{j=n}\kappa_{ij}$$

The credibility degree represents the credibility of evidence, which is highly correlated with its similarity. The higher the similarity between evidence and others, the more credible the evidence is. The credibility of the evidence is denoted as follows:

(11) $$\psi_{cr}\left({ℵ}_{i}\right)=\frac{\psi_{sup}\left({ℵ}_{i}\right)}{\sum_{j}\psi_{sup}\left({ℵ}_{j}\right)}$$

In this paper, the credibility of evidence can be viewed as its weight, which can be denoted as follows:

(12) $$weight_{i}=\psi_{cr}\left({ℵ}_{i}\right)$$

In attribute reduction, there is a reciprocal and symmetrical relationship between attributes. In information theory, symmetric uncertainty can be utilized to measure the information contained in attributes. Therefore, we define the quality function (mass) of evidence using symmetric uncertainty.

**Definition** **7.** ***Symmetric Uncertainty:** Given ${ℜ}_{DS}=\left(U,C,D,\nu \right)$, for ∀B⊆C, the symmetric uncertainty between B and D is defined as follows:*

(13) $${ℑ}_{SU}\left(B,D\right)=\frac{2{ℑ}_{MI}\left(B,D\right)}{{ℑ}_{E}\left(B\right)+{ℑ}_{E}\left(D\right)}$$

From Equation (13), it can be seen that a larger value indicates a higher correlation between the two variables, resulting in a higher weight ratio and vice versa. Compared with mutual information, symmetrical uncertainty can quantify the symmetry between different pieces of evidence, which is the correlation between attributes and labels and the dependence between labels and attributes.

Given multiple attribute subsets as input, we adopt evidence fusion theory to obtain a global attribute subset. For ∀a∈C, the fusion rule is defined as follows:

(14) $$\left({ℵ}_{1}⊕{ℵ}_{2}⊕\cdots ⊕{ℵ}_{n}\right)\left(a\right)=\frac{1}{K}\prod_{i=1}^{n}{ℵ}_{i}\left(a\right)$$

where $K=\sum_{i=1}^{i=|C|}\prod_{j=1}^{n}{ℵ}_{j}\left(i\right)$ is the conflict constant between the pieces of evidence.

The pseudo-code for the evidence fusion process is shown in Algorithm 2.
**Algorithm 2** Evidence Fusion.**Input**: Evidence set. **Output**: fusion result ${ℵ}_{best}$.   1: ${ℵ}_{best}\leftarrow \emptyset$   2: calculate the mass function of each evidence by Equation (13),   3: calculate the conflict matrix using Equations (6) and (7)   4: calculate the similarity matrix using Equation (9)   5: calculate the support function of each evidence using Equation (10)   6: calculate the credibility of each evidence using Equation (11)   7: calculate the fusion result ${ℵ}_{best}$ using Equation (14)   8: return to the fusion best result ${ℵ}_{best}$

For Algorithm 2, it is assumed that the number of samples is $\left|U\right|$, there are $\left|N\right|$ pieces of evidence, and the number of independent attributes in each piece of evidence is $\left|C\right|$. Then, the time complexity of calculating the quality function is $O\left(\left|U\right|\right)$, the time complexity of calculating the similarity matrix is $O(|N||C{|}^{2})$, the time complexity of calculating the credibility process of the evidence is O(|N||C|), and the time complexity of fusing evidence is $O(|N||C{|}^{2})$. Therefore, the time complexity of Algorithm 2 is $O(\max(|N||C{|}^{2},|U|))$.

Illustrate the process of evidence fusion theory in a more intuitive way through an example.

**Example** **2.** *The decision information system is the same as that in Example 1. Now, suppose there are three pieces of evidence ${ℵ}_{a}=\left\{a_{1},a_{2},a_{3},a_{4}\right\},{ℵ}_{b}=\left\{a_{2},a_{4}\right\},{ℵ}_{c}=\left\{a_{1},a_{2},a_{3}\right\}$, and the evidence fusion process is as follows.*

To simplify the calculation, it is assumed that ω=2 in Formula (6). It is easy to obtain $\chi_{ab}=0.6634$, $\chi_{ac}=0.5452$, $\chi_{bc}=0.8203$. Therefore, the evidence conflict matrix can be expressed as follows.

$$MC=\left[\begin{array}{ccc} 0 & 0.6634 & 0.5452\\ 0.6634 & 0 & 0.8203\\ 0.5452 & 0.8203 & 0 \end{array}\right]$$

Furthermore, its similarity matrix can be expressed as follows:

$$MS=\left[\begin{array}{ccc} 1 & 0.3366 & 0.4548\\ 0.3366 & 1 & 0.1797\\ 0.4548 & 0.1797 & 1 \end{array}\right]$$

According to the similarity matrix, we have

$$\psi_{sup}\left({ℵ}_{a}\right)=0.7914,\psi_{sup}\left({ℵ}_{b}\right)=0.5163,\psi_{sup}\left({ℵ}_{c}\right)=0.6345$$

So,

$$\psi_{cr}\left({ℵ}_{a}\right)=0.4074,\psi_{cr}\left({ℵ}_{b}\right)=0.2658,\psi_{cr}\left({ℵ}_{c}\right)=0.3267$$

According to Equations (12) and (13), the corresponding weights can be obtained. Now, the evidence is

$${ℵ}_{a}=\left\{0.1976,0.1735,0.1843,0.2221\right\},{ℵ}_{b}=\left\{0.1132,0.1449\right\},{ℵ}_{c}=\left\{0.1585,0.1391,0.1478\right\}$$

Accordingly, it is known that K=0.021; the fusion result is

$${ℵ}_{best}=\left\{16.95,21.20,15.40,10.72\right\}$$

Up to this point, the evidence fusion process is completed.

### 4.2. IILE Model

The output of Algorithm 2 is a set of weight-ordered attributes, where each element represents the weight of an attribute after fusion. The higher the weight, the higher the priority of the attribute.

It should be noted that, in practical applications, the obtained dataset often contains a certain number of noise samples. Noise samples affect the reduction effect, thus affecting the classification performance. Therefore, based on Algorithms 1 and 2, we introduce the approximate reduction approach to obtain Algorithm 3 (IILE), which can consider the classification ability and globality so that it can be immune to the interference of some noise samples. The pseudo-code for the evidence fusion process is shown in Algorithm 3.

For Algorithm 3, let us consider the number of voters (the number of attribute reduction processes). Each voter randomly samples from the original dataset to construct an independent sample subspace for attribute reduction and stores it in the reduction set to be fused. Then, multiple attribute subsets are fused to obtain a weight order using the evidence fusion theory. During the positive selection of attributes, the attributes with higher weight are preferred. Finally, the number of inconsistent samples is utilized as the end marker for positive selection. In the dataset, the classification ability of the attribute subset is mainly affected by the inconsistent samples. The greater the number of inconsistent samples, the weaker the classification ability of the attribute subset.
**Algorithm 3** Attribute reduction based on incremental information level and group optimization strategy (IILE).**Input**: ${ℜ}_{DS}=\left(U,C,D,\nu \right)$. **Output**: reduction set red.
   1:ϑ←∅, vm←n   2:$tred_{i}\leftarrow$ randomly sampling from ${ℜ}_{DS}$ using Algorithm 1, $\vartheta \leftarrow \vartheta \cup \left\{tred_{i}\right\}$   3:${ℵ}_{best}\leftarrow \vartheta$ using Algorithm 2   4:$\delta_{C}\leftarrow$ calculate the number of inconsistent samples of *C*   5:select attribute $a_{0}$ with the highest weight in ${ℵ}_{best}$, and then set its weight equal to 0   6:$red\leftarrow red\cup \left\{a_{0}\right\}$   7:calculate the number of inconsistent samples $\delta_{red}$, if $\delta_{red}>\beta *\delta_{C}$, go to line 8, otherwise go to line 5   8:return to the reduction red.

## 5. Experiments

To better verify the performance of IILE, we conducted multiple experiments on eight datasets of different sizes from the University of California Irvine (UCI) and Arizona State University (ASU). The details of the dataset are shown in Table 3 (|C| represents the number of attributes in the dataset, |U/D| denotes the number of decision attributes, and |U/C| indicates the number of equivalence classes divided based on attributes).

A detailed description of the above-mentioned dataset in the practical fields is as follows:

- CHES: It is a chess endgame dataset for machine learning research related to predicting the outcomes of chess endgames;
- LEK: It is used to identify normal cells from leukemia blast cells, assisting doctors in diagnosing leukemia more accurately;
- SPLI: It aims to study and identify the boundaries between introns and exons in DNA sequences to facilitate the understanding of the DNA splicing process in the field of molecular biology;
- SPEC: It is a medical dataset mainly with regard to cardiology for diagnosing heart diseases;
- PROM: It is employed for the analysis of transcription factor binding sites and the study of gene expression regulation mechanisms in the field of gene expression regulation;
- LYMP: It is a dataset on lymphography research from the University Medical Centre, Institute of Oncology, Ljubljana, Yugoslavia;
- COLON: It is a dataset that focuses on the research of colon diseases to assist in treatment;
- WAVE: It is a waveform dataset used in physics.

In this section, we describe ablation experiments that we performed between IILE and IIL to assess the feasibility of evidence fusion theory. Second, to evaluate the effectiveness of IILE, we executed contrast experiments with other attribute reduction approaches. Finally, we introduced statistical analysis [36] to further demonstrate the performance of IILE in a statistical context.

### 5.1. Experimental Setup

To verify the effectiveness of IILE, we performed a comparative analysis with several approaches:

- HS [22]: They proposed a reduction algorithm of neighborhood rough set based on hypersphere to optimize the extraction of equivalence relation;
- FSFC [24]: They evaluated features from the global feature correlation and local feature correlation to improve the classification ability of the selected attributes;
- GNRS [27]: They presented a parameter-free rough set method based on a random clustering center to adjust the neighborhood radius adaptively;
- SRS [28]: They designed a more concise attribute reduction algorithm based on the principle of spatial similarity;
- PME [31]: They converted a multi-valued classification into a binary classification problem and set different weight parameters for two classes;
- MDS [37]: They proposed a method for integrating multiple model reduction results using evidence theory.

To intuitively demonstrate the accuracy differences, we adopted multiple classifier models for comparison in the experiment, including the *K* nearest neighbor classifier (KNN), support vector machine (SVM), and CART decision tree (CART). In addition, the F1 score was utilized to validate the performance of our approach against others. Meanwhile, to ensure the fairness of the comparison algorithm, all the parameters were set to the defaults recommended by the original paper.

### 5.2. Ablation Experiment

**Figure 2:**
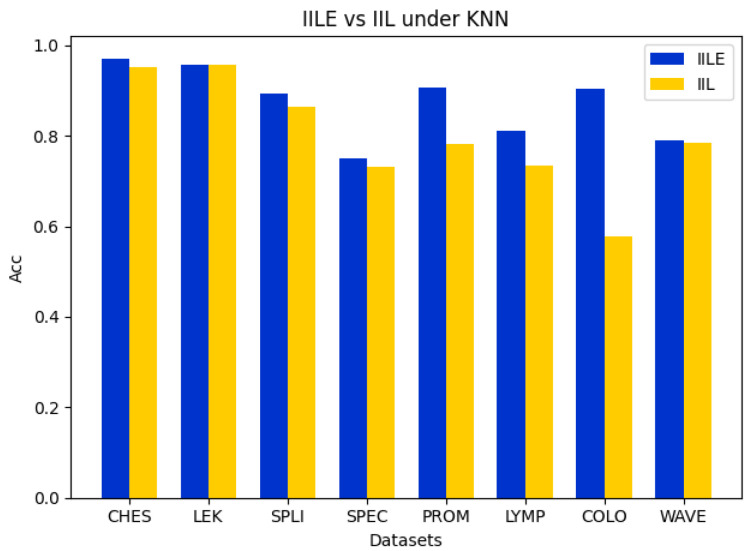
Comparison of accuracy between IILE and IIL on KNN.

**Figure 3:**
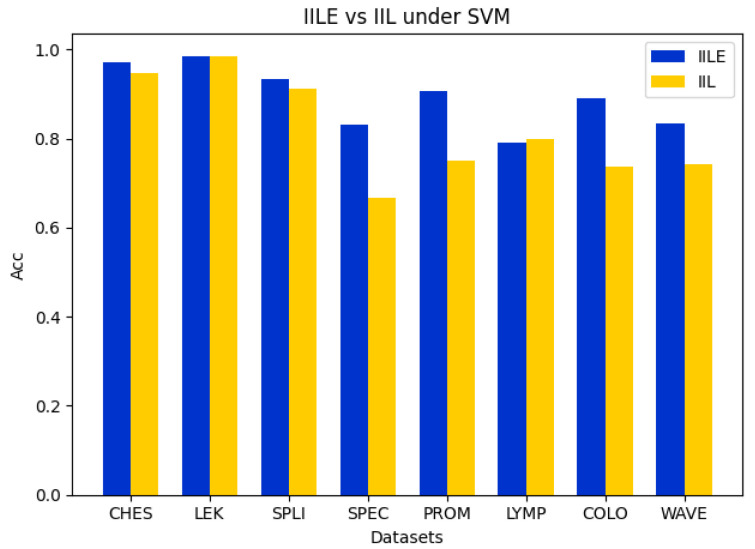
Comparison of accuracy between IILE and IIL on SVM.

**Figure 4:**
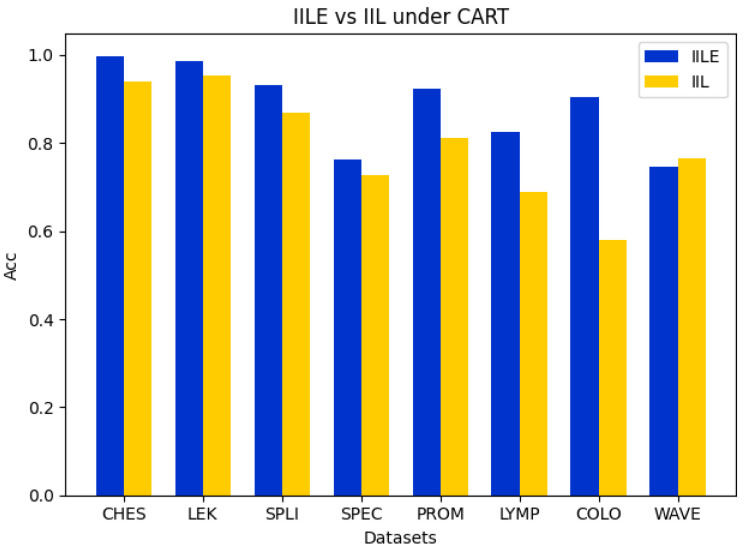
Comparison of accuracy between IILE and IIL on CART.

**Figure 5:**
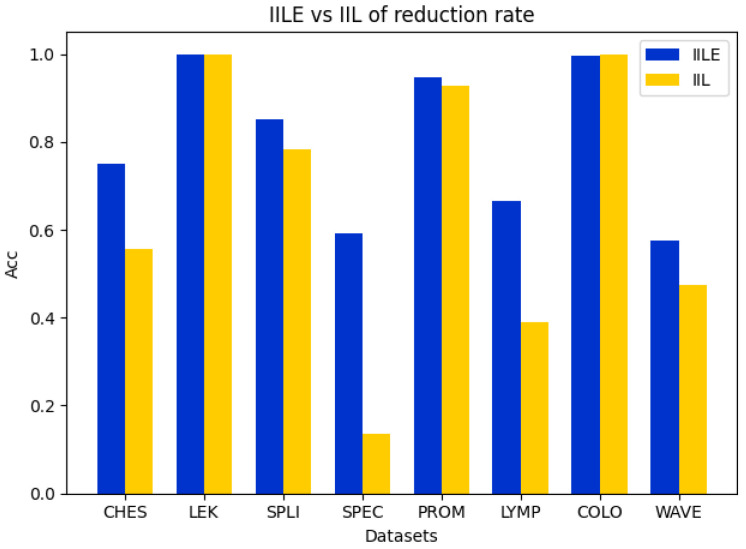
Comparison of reduced rate between IILE and IIL.

In this subsection, we perform the comparison ablation experiments to demonstrate the effectiveness of the evidence fusion between IILE and IIL. For IIL, it directly executes reduction for a data subspace by sampling from the original data and then trains the classification model to assess the algorithm’s accuracy and reduction rate. For IILE, the original data are randomly sampled several times to construct multiple data subspaces. Then, a reduction process in each data subspace is performed to obtain the reduction subsets. Finally, the evidence fusion strategy is utilized to obtain the final reduction subset to characterize the original feature space, and the classification model is further trained to evaluate the algorithm’s accuracy and reduction rate. The details of the experiments are shown in Figure 2, Figure 3, Figure 4 and Figure 5.

In Figure 2, Figure 3 and Figure 4, it can be seen that the difference between the IILE and IIL results is most significant for the COLO dataset. The IILE result is 32.59% higher than the IIL result with KNN and CART and 15.37% higher than that with the SVM. The reduction rate of the COLO dataset is shown in Figure 5. We can see that the IIL reduction number is 5, with a reduction rate of 99.75%, and that the IILE reduction number is 8, with a reduction rate of 99.60%. The results highlight the impact of noise data on IIL during the attribute extraction process. Only the correlation between the attributes and labels is considered when determining whether the reduction results are generated or not, leading to an inaccurate assessment of the current attribute reduction’s classification capability. For IILE, the evidence fusion strategy was adopted to improve the classification accuracy by fusing multiple IIL rounds’ outcomes. Based on the data shown in Figure 2, Figure 3 and Figure 4, the IILE’s classification accuracy using evidence fusion theory exceeds that of IIL. To sum up, the evidence fusion strategy in our proposal can enhance both the reduction rate and the reduction’s capability for classification.

In order to demonstrate the advantages of our proposal more specifically, we take the LYMP dataset as an example to illustrate the differences in their classification capabilities through classification accuracy (the ratio of the number of correctly classified samples to the total number of samples, ACC=Correct/ALL).

Suppose the number of evidence is 6, and its reduction result is [12, 16, 13, 17, 1, 14], [17, 12, 13, 1, 9, 14], [12, 13, 17, 1, 14, 9, 11], [12, 17, 13, 1, 9, 14, 11], [17, 13, 12, 1, 9, 14, 4, 0], [12, 17, 1, 14, 13, 9, 11, 0, 16, 4], according to the above formula, their accuracies are 0.73333, 0.7467, 0.7155, 0.7435, 0.7570 and 0.7311 respectively. The result of fusion through the evidence fusion theory is [12, 17, 1, 14, 13], and its classification accuracy is 0.7800. Obviously, while the number of attributes has been significantly reduced, the classification ability has been improved, which intuitively demonstrates the effectiveness of evidence theory.

### 5.3. Effectiveness Experiment

The above ablation experiments have already validated that IILE outperformed IIL overall. Therefore, we perform contrast experiments to assess the effectiveness performance of IILE compared with other algorithms in this subsection. The specific experimental results are presented in Table 4, Table 5 and Table 6.

The comparison accuracies of different approaches under the KNN are detailed in Table 4. It shows that the accuracy of IILE on the PROM dataset was 90.64%, which is a 33.82% increase compared to SRS. In addition, for SRS, the COLON dataset has 63 samples and 2000 attributes, making it hard to extract comparable attributes and resulting in unsatisfactory performance. On the SPEC dataset, the performance of IILE is inferior to that of SRS and PME. The performance of the MDS algorithm is comparable to that of PME, but there is still a certain gap compared to IILE. Considering all the above factors, the average classification accuracy of IILE is 87.30%, representing a 3.72% improvement over PME.

Table 5 mainly explains the comparison results of HS, FSFC, GNRS, SRS, PME, MDS, and IILE on the SVM. From Table 5, it can be seen that the reductions obtained by IILE can obtain high classification accuracy on CHES, LEK, SPLI, PROM, and WAVE. Although our proposal is not the best on SPEC, LYMP, and COLON, the gap between its performance and the best results is all less than 1%. Based on the overall analysis, IILE has better classification accuracy than the others on most of the datasets.

Table 6 presents the classification accuracies of HS, FSFC, GNRS, SRS, PME, MDS, and IILE on the CART. The results show that IILE can obtain the best results on most datasets except with LEK, SPEC, and WAVE. PME achieved the best results on the LEK and SPEC sets, while SRS achieved the best on WAVE. On LEK, SPEC, and WAVE, IILE is not optimal, but the difference between its performance and the best results is almost negligible. On the datasets PROM and LYMP, IILE can achieve the best classification accuracy, and the results increased by 4.9% and 4.72% compared with those of the sub-optimal algorithm, respectively. On average, the performance of MDS is similar to that of PME, and the difference between the two is within 1%. The above experiments intuitively show the classification accuracy of IILE.

Table 7 presents the reduction rates for HS, FSFC, GNRS, SRS, PME, MDS, and IILE. IILE achieves the highest reduction rate on four datasets (LEK, SPLI, PROM, and LYMP). Although IILE performed worse than PME on CHES, IILE outperformed PME on KNN, SVM, and CART by 8.15%, 2.55%, and 0.5%, respectively. For MDS, it exhibits the best reduction ability on the COLON dataset. However, when considering Table 4 and Table 5 comprehensively, the overall classification ability of MDS has declined slightly compared with that of PME and IILE. To sum up, the average reduction efficiency of IILE was 79.68%, which could generate an effective reduction for the dataset, and the generated reduction could lead to better classification ability.

Table 8, Table 9 and Table 10 present the F1scores of HS, FSFC, GNRS, SRS, PME, MDS, and IILE under KNN, SVM, and CART, respectively. The results demonstrate that IILE performed excellently. It achieves the best F1 scores in KNN for PROM, SVM for LEK, and CART for CHES and LEK. The results in Table 8, Table 9 and Table 10 indicate that IILE has a high classification accuracy and a superior recall rate. Even though our proposed method could not obtain the highest F1score on a few datasets, it consistently obtained the highest score overall. Therefore, the experimental results further demonstrate that IILE exhibits superior performance.

The above experimental results show that IILE can obtain the optimal results on most datasets. It is only slightly worse than optimal on very few specific datasets. Therefore, the effectiveness of IILE is fully verified by combining the results of multiple classifier models.

### 5.4. Application Experiments

In the previous section, we have already demonstrated the effectiveness of IILE. To verify the operability of IILE in specific fields, an analysis is performed in this section on the medical, biological, and physics datasets. The dataset information is shown in Table 11 (the information represented by each column is the same as that in Table 3).

The LUNG dataset in the medical field is utilized to discriminate whether a patient has lung cancer or not. It includes 15 indicators, such as gender, age, whether the patient has a chronic disease, etc. However, in the unprocessed dataset, it is unclear whether redundant attributes exist or not affect the judgment and which subset of attributes strongly correlate with the judgment of lung cancer. In order to validate the effectiveness of our proposal, we conducted cross-training on unprocessed attributes and trained by KNN, SVM, and CART, respectively. The results are 0.8897, 0.906, and 0.8898. Then, we employ IILE to process the LUNG dataset. The reduction result is [8, 10, 13, 11, 4, 9, 3, 5, 14, 1, 7], with a dimension of 11. The classification accuracies trained by KNN, SVM, and CART are 0.8898, 0.9094, and 0.9253, respectively. Compared with unprocessed attributes, the classification capabilities are significantly improved. The above results indicate that there exist redundant attributes in the original dataset. These redundant attributes can impact the classifier’s training process, thus reducing the classification accuracy. In addition, using the reduced attribute sequence for diagnosis can reduce patients’ medical costs. Meanwhile, it can enhance the efficiency and accuracy of medical consultations.

The MUSH dataset in the biological field is used to judge the species of mushrooms based on the appearance of different parts of the mushrooms. The unprocessed dataset contains 22 attributes. Similarly, a cross-training is performed on the original attributes trained by KNN, SVM, and CART, respectively, and the results are 1, 0.9997, and 1, respectively. Then, we adopt the same strategy for cross-validation. However, the difference is that the data attributes are processed using IILE, and the reduction result obtained was [4, 17, 7, 18], corresponding to four attributes: odor, gill size, ring type, and ring number. The classification accuracies of KNN, SVM, and CART are 0.9887, 0.9872, and 0.9911, respectively. The above results indicate that for mushroom classification, an accuracy of 98% can be achieved by using only 18.1% of the attributes in the original dataset after attribute reduction.

The SF dataset from the physics and chemistry field contains 12 attributes to count the number of solar flares of a certain class that occur in a 24 h period. Firstly, cross-training is performed on the original attributes using KNN, SVM, and CART. The corresponding results are 0.6614, 0.7393, and 0.6835, respectively. Then, we conduct a reduction by using IILE and the length of the reduced result is 10. The classification accuracies of KNN, SVM, and CART are 0.6833, 0.7393, and 0.6927, respectively. It can be seen that IILE can perform effective reduction when the quality of data is terrible, eliminating the interference of low-correlation attributes for workers and improving the accuracy of classification tasks simultaneously.

To sum up, the above results indicate that IILE is both operable and interpretable in practice, and the effectiveness of its reduction results can be verified in practical applications.

### 5.5. Statistical Analysis

The above experiments fully demonstrated the effectiveness of IILE. This section describes further experiments conducted using the Friedman test [36]. The Friedman test uses the ranking of each algorithm on different datasets to verify its statistical significance. After conducting the Friedman test, its test statistic is compared with the chi-square distribution to verify the null hypothesis. The results of the Friedman test characterize the performance of different reduction methods. The smaller the value, the better the stability and performance. The formula for the Friedman test is as follows:

(15) $$\Lambda_{F}^{2}=\frac{12N}{k(k+1)}\left[\sum_{j}R_{j}^{2}-\frac{k{\left(k+1\right)}^{2}}{4}\right]$$

where *k* represents the number of approximation algorithms, *N* represents the number of datasets, $r_{i}^{j}$ represents the *j*-th algorithm of *k* algorithms, $R_{j}=\sum_{i}r_{i}^{j}$.

The results of the Friedman test are shown in Table 12.

In Table 12, it can be easily observed that, among the KNN, SVM, and CART classifiers, the value of IILE is the smallest, and IILE also has the smallest value among the average results. The experiment results indicate significant differences between IILE and other algorithms.

However, the Friedman test can only prove significant differences between the algorithms mentioned above and cannot determine which algorithms have significant differences. Therefore, the Nemenyi test is performed, and a critical graph is provided to visually display the differences between the algorithms. If the difference between the two algorithms is greater than the CD (critical difference) value, the difference is significant. The formula for calculating the CD value is as follows:

(16) $$CD=q_{\alpha }\sqrt{\frac{k(k+1)}{6N}}$$

where *k* represents the number of algorithms and *N* represents the number of datasets.

**Figure 6:**
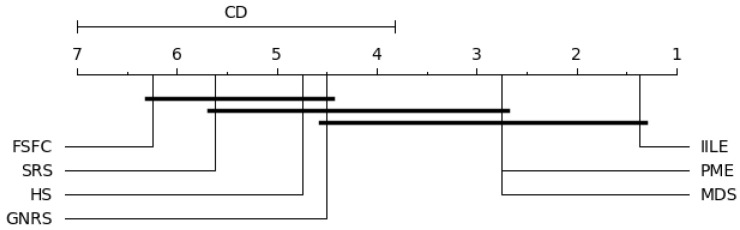
KNN critical difference graph.

**Figure 7:**
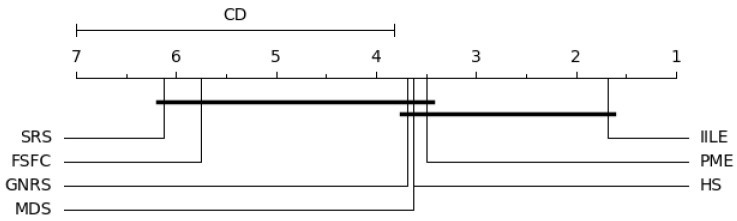
SVM critical difference graph.

**Figure 8:**
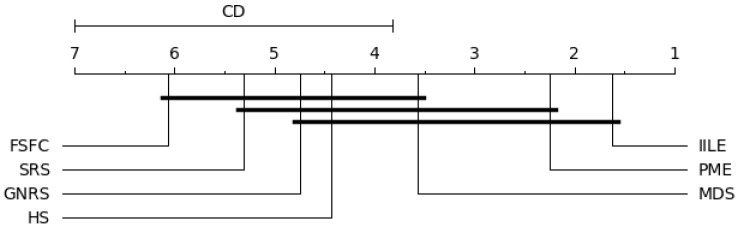
CART critical difference graph.

**Figure 9:**
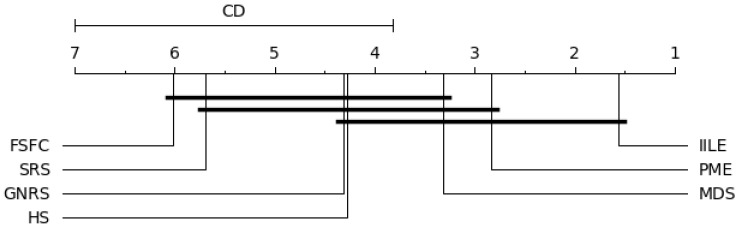
Average critical difference graph.

In our experiments, when k=7 and N=8, $q_{\alpha }=2.949$, the level of significance is α=0.05; the results are shown in Figure 6, Figure 7, Figure 8 and Figure 9.

Figure 6 shows significant differences between IILE and HS, FSFC, SRS, GNRS under KNN, PME performs consistently with MDS, and both of them are superior to FSFC and SRS. For SVM (as shown in Figure 7), a significant difference exists between IILE and SRS, FSFC, GNRS, PME has a slight difference with MDS. Figure 8 shows significant differences between IILE and FSFC, SRS, and GNRS under CART. It is worth noting that under the CART classification model, the difference between PME and MDS has expanded, although this is not significant (the difference is less than the CD value). The average results in Figure 9 show significant differences between IILE, FSFC, GNRS, and SRS. The above results statistically indicate that IILE is superior to the other methods.

## 6. Conclusions

Nowadays, attribute reduction is one of the research hot spots in practical applications. Therefore, in this paper, we present a novel ensemble attribute reduction algorithm based on an incremental information level and improved evidence theory in order to obtain a more relevant attribute set. Firstly, an incremental mutual information level between attributes and labels is proposed for judging the optimal attribute. Then, we further utilize the improved evidence fusion strategy for obtaining the most representative attribute subset in order to enhance the robustness of the model. Additionally, we utilize approximate reduction in evidence fusion theory to minimize the impact of noise data on classification performance. The experimental results further confirm the effectiveness of our proposed algorithm.

In our future work, the following aspects can continue to be improved:

1. The weight of the two parts of the incremental information level may differ for different datasets. Therefore, we consider setting the appropriate weights as being beneficial for attribute reduction.
2. The weight value for evidence fusion theory is crucial. The evidence theory can combine multiple results to produce a more robust attribute subset, but this greatly depends on the weight of each piece of evidence. Therefore, we will focus on how to set the weight more reasonably.

## Figures and Tables

**Table 1:** Comparison between different algorithms.

Model	Correlation	Redundancy	Approximate Reduction	Ensemble Learning
WDN	✓	×	×	×
HS	✓	✓	×	×
WKN	✓	×	×	×
FSCE	✓	×	×	×
GBNRS	✓	×	✓	×
SRS	✓	✓	✓	×
PME	✓	✓	×	×
NSI	✓	✓	×	×
FSR	✓	✓	×	×
IILE	✓	✓	✓	✓

**Table 2:** Decision information system ${ℜ}_{DS}=\left(U,C,D,\nu \right)$.

	$c_{1}$	$c_{2}$	$c_{3}$	$c_{4}$	*d*
$u_{1}$	0	0	0	0	$d_{1}$
$u_{2}$	0	0	0	1	$d_{2}$
$u_{3}$	0	0	0	1	$d_{2}$
$u_{4}$	0	0	1	1	$d_{2}$
$u_{5}$	0	0	0	1	$d_{2}$
$u_{6}$	0	0	0	1	$d_{1}$
$u_{7}$	0	1	1	1	$d_{2}$
$u_{8}$	0	1	1	1	$d_{3}$
$u_{9}$	0	1	1	1	$d_{3}$
$u_{10}$	1	1	1	1	$d_{1}$
$u_{11}$	1	1	1	1	$d_{3}$

**Table 3:** The details of the dataset.

Dataset	Abbreviation	Sample	|C|	|U/D|	|U/C|	Sources
chess	CHES	3196	36	2	7	UCI
leukemia	LEK	72	7070	2	3	ASU
splice	SPLI	3175	60	3	4	UCI
spect	SPEC	267	22	2	2	UCI
promoters	PROM	105	56	2	4	UCI
lymphography	LYMP	148	18	4	8	UCI
colon	COLON	62	2000	2	3	ASU
waveform	WAVE	5000	40	3	3	UCI

**Table 4:** Comparison of KNN classification accuracy of different algorithms.

Dataset	HS	FSFC	GNRS	SRS	PME	MDS	IILE
CHES	0.9218	0.5369	0.9230	0.6608	0.8899	0.9090	**0.9714**
LEK	0.9000	0.6143	0.8333	0.6964	0.9240	0.9122	**0.9571**
SPLI	0.6875	0.6891	0.6948	0.5049	0.8652	0.8671	**0.8926**
SPEC	0.7328	0.7308	0.7415	0.7561	**0.7566**	0.7521	0.7490
PROM	0.8491	0.5818	0.6646	0.5682	0.8973	0.8838	**0.9064**
LYMP	0.7419	0.7305	0.7576	0.6348	0.7924	0.8015	**0.8119**
COLON	0.7071	0.6619	0.5310	0.4833	0.8429	0.8316	**0.9048**
WAVE	0.6651	0.4673	0.6967	0.7562	0.7184	0.7854	**0.7908**
Average	0.7756	0.6266	0.7303	0.6326	0.8358	0.8428	**0.8730**
Rank	4	7	5	6	3	2	1

• Note that each dataset’s best result is highlighted in bold. The ‘Rank’ row denotes the algorithm’s ranking. The ‘Average’ row represents the average classification accuracy for each approach.

**Table 5:** Comparison of SVM classification accuracy of different algorithms.

Dataset	HS	FSFC	GNRS	SRS	PME	MDS	IILE
CHES	0.9587	0.5882	0.9546	0.7078	0.9459	0.9558	**0.9714 **
LEK	0.8732	0.6554	0.7917	0.7500	0.9568	0.9271	**0.9587**
SPLI	0.9020	0.7436	0.9040	0.5427	0.9153	0.9047	**0.9339**
SPEC	0.8382	0.7793	**0.8388**	0.8057	0.8197	0.8288	0.8312
PROM	0.8282	0.5509	0.7836	0.5955	0.8964	0.8791	**0.9064**
LYMP	0.7962	**0.8048**	0.7905	0.7038	0.7705	0.7855	0.7905
COLON	0.7714	0.7333	0.6476	0.5976	**0.9014**	0.8516	0.8905
WAVE	0.5093	0.5281	0.8228	0.7954	0.7846	0.8002	**0.8334**
Average	0.8096	0.6730	0.8167	0.6873	0.8738	0.8660	**0.8929**
Rank	5	7	4	6	2	3	1

• Note that each dataset’s best result is highlighted in bold. The ‘Rank’ row denotes the algorithm’s ranking. The ‘Average’ row represents the average classification accuracy for each approach.

**Table 6:** Comparison of CART classification accuracy of different algorithms.

Dataset	HS	FSFC	GNRS	SRS	PME	MDS	IILE
CHES	0.9693	0.5882	0.9850	0.6940	0.9931	0.9875	**0.9981 **
LEK	0.9750	0.6789	0.8472	0.6786	**0.9928**	0.9714	0.9857
SPLI	0.9150	0.7099	0.9118	0.5373	0.9156	0.9150	**0.9313**
SPEC	0.7511	0.7793	**0.8388**	0.8057	0.8197	0.7422	0.8312
PROM	0.7309	0.5800	0.7236	0.5209	0.8755	0.8571	**0.9245**
LYMP	0.7495	0.7157	0.7148	0.6352	0.7776	0.7621	**0.8248**
COLON	0.7381	0.7238	0.5643	0.6595	0.7786	0.7920	**0.9048**
WAVE	0.5029	0.5235	0.7228	**0.7504**	0.7016	0.7116	0.7465
Average	0.7915	0.6558	0.7795	0.6555	0.8512	0.8424	**0.8850**
Rank	4	7	5	6	2	3	1

• Note that each dataset’s best result is highlighted in bold. The ‘Rank’ row denotes the algorithm’s ranking. The ‘Average’ row represents the average classification accuracy for each approach.

**Table 7:** Comparison of reduced rate of different algorithms.

Dataset	HS	FSFC	GNRS	SRS	PME	MDS	IILE
CHES	0.0556	0.9444	0.0556	0.7500	**0.9997**	0.7500	0.7500
LEK	0.1667	0.9992	0.9993	0.9990	0.1667	0.9987	**0.9996**
SPLI	0.0167	0.8667	0.0167	**0.8500**	0.8167	0.8320	**0.8500**
SPEC	0.2273	0.5455	0.2727	0.5909	0.1818	0.2727	0.5909
PROM	0.7857	0.8929	0.7857	0.8929	0.9286	0.9286	**0.9464**
LYMP	0.3889	**0.6667**	0.1667	0.5556	0.6667	0.5842	**0.6667**
COLON	0.9935	0.9960	0.9935	0.9955	0.9980	**0.9987**	0.9960
WAVE	**0.9000**	**0.9000**	0.0500	0.7750	0.6250	0.5951	0.5750
Average	0.4418	**0.8514**	0.4175	0.8011	0.6729	0.7450	0.7968
Rank	7	1	6	2	5	4	3

• Note that each dataset’s best result is highlighted in bold. The ‘Rank’ row denotes the algorithm’s ranking. The ‘Average’ row represents the average classification accuracy for each approach.

**Table 8:** Comparison of KNN F1-score with different algorithms.

Dataset	HS	FSFC	GNRS	SRS	PME	MDS	IILE
CHES	**0.9500**	0.5933	0.9135	0.6611	0.9197	0.9011	0.9091
LEK	0.9091	0.6818	0.8114	0.6364	**0.9510**	0.8556	0.8636
SPLI	0.6684	0.7030	0.6548	0.5100	0.8573	0.7542	**0.9129**
SPEC	0.8125	0.7407	0.7284	0.7654	0.7901	0.7433	**0.8395**
PROM	0.8125	0.5625	0.6875	0.5313	**1.0000**	0.8948	**1.0000**
LYMP	0.7556	0.7556	0.7778	0.6889	0.7556	0.8069	**0.8222**
COLON	0.5790	0.5790	0.5790	0.5263	0.8421	0.8421	**0.9474**
WAVE	0.4553	0.4927	0.7033	0.7553	0.7153	0.7454	**0.7840**
Average	0.7428	0.6386	0.7319	0.6343	0.8539	0.8179	**0.8848**
Rank	4	6	5	7	2	3	1

• Note that each dataset’s best result is highlighted in bold. The ‘Rank’ row denotes the algorithm’s ranking. The ‘Average’ row represents the average classification accuracy for each approach.

**Table 9:** Comparison of SVM F1-score with different algorithms.

Dataset	HS	FSFC	GNRS	SRS	PME	MDS	IILE
CHES	0.9635	0.5933	0.9500	0.7080	**1.0000**	0.9500	0.9416
LEK	**1.0000**	0.6364	0.7787	0.6364	0.9520	0.8948	**1.0000**
SPLI	0.8982	0.7671	0.8919	0.5509	0.9108	0.9027	**0.9265**
SPEC	0.8000	0.7531	0.8025	0.7531	0.7901	0.8613	**0.8765**
PROM	0.7813	0.6250	0.7813	0.5625	0.9688	0.9483	**0.9688**
LYMP	0.7778	0.7556	0.7333	**0.8000**	**0.8000**	0.7778	**0.8000**
COLON	0.5263	0.7368	0.5263	0.6316	**1.0000**	0.7782	0.8947
WAVE	0.5153	0.5393	0.8127	0.7927	0.7827	0.7797	**0.8360**
Average	0.7828	0.6758	**0.7846**	0.6794	0.9005	0.8616	**0.9055**
Rank	5	7	4	6	2	3	1

• Note that each dataset’s best result is highlighted in bold. The ‘Rank’ row denotes the algorithm’s ranking. The ‘Average’ row represents the average classification accuracy for each approach.

**Table 10:** Comparison of CART F1-score with different algorithms.

Dataset	HS	FSFC	GNRS	SRS	PME	MDS	IILE
CHES	0.9604	0.5933	0.9771	0.7049	0.9937	0.9877	**1.0000**
LEK	0.9546	0.6364	0.8025	0.6364	0.9896	0.9654	**1.0000**
SPLI	0.8951	0.7062	0.9014	0.5331	0.8972	0.8862	**0.9150**
SPEC	**0.7875**	0.7037	0.7407	0.7037	0.7037	0.7407	0.7037
PROM	0.6563	0.6875	0.6875	0.4375	**0.9375**	0.8241	0.9063
LYMP	0.7333	0.7111	0.7111	0.7111	**0.7556**	0.7556	**0.7556**
COLON	**0.6316**	0.7368	0.6316	0.6316	**0.8421**	0.7368	**0.8421**
WAVE	0.5147	0.5240	0.7000	0.7347	0.5913	0.7233	**0.7433**
Average	0.7667	0.6624	0.7690	0.6366	0.8513	0.8275	**0.8582**
Rank	5	6	4	7	2	3	1

• Note that each dataset’s best result is highlighted in bold. The ‘Rank’ row denotes the algorithm’s ranking. The ‘Average’ row represents the average classification accuracy for each approach.

**Table 11:** The details of the dataset.

Dataset	Abbreviation	Sample	|C|	|U/D|	|U/C|	Sources
lung cancer	LUNG	3196	15	2	181	UCI
mushrooms	MUSH	8125	22	2	2249	UCI
solar-flare	SF	323	12	2	158	UCI

**Table 12:** Comparison of Friedman test under different classifiers.

Algorithm	KNN	SVM	CART	Average
HS	4.7500	3.6250	4.4375	4.2708
FSFC	6.2500	5.7500	6.0625	6.0208
GNRS	4.5000	3.6875	4.7500	4.3125
SRS	5.6250	6.1250	5.3125	5.6875
PME	2.7500	3.5000	2.2500	2.8333
MDS	2.7500	3.6250	3.5625	3.3125
IILE	1.3750	1.6875	1.6250	1.5625

• Note that KNN *p*-value is 0.001, SVM *p*-value is 0.001, and CART *p*-value is 0.001.

## Data Availability

The data used to support the findings of this study are included in the article.
